# The relationship between ablation range and ablation energy in papillary thyroid microcarcinoma: a comparison between microwave ablation and laser ablation

**DOI:** 10.1007/s00330-024-10636-4

**Published:** 2024-02-10

**Authors:** Xinyu Zhong, Yuting Cao, Xinghao Zhang, Wengang Liu, Ping Zhou

**Affiliations:** 1 Department of Ultrasonography, The Third Xiangya Hospital, Central South University, No.138 Tongzipo Road, Changsha, 410013 Hunan China; 2 Institute of Ultrasound Imaging, The Second Affiliated Hospital of Chongqing Medical University, Chongqing, 400010 China

**Keywords:** Papillary thyroid microcarcinoma, Ultrasonography, Microwaves, Laser therapy, Ablation techniques

## Abstract

**Objectives:**

To study the relationship between the ablation range and applied energy of laser ablation (LA) and microwave ablation (MWA) in papillary thyroid microcarcinoma (PTMC).

**Methods:**

A total of 201 PTMC patients were treated with LA (*n* = 102) or MWA (*n* = 99) with single-applicator fixed ablation. The ablation range was determined by contrast-enhanced ultrasound. The ratios of ablation volume, longitudinal diameter, and orthogonal diameter to ablation energy (R_AV/E_, R_AL/E_, R_AO/E_) were analyzed and compared between MWA and LA. The effects of PTMC characteristics and Hashimoto’s thyroiditis (HT) on ablation efficiency were evaluated by linear regression.

**Results:**

The R_AV/E_ was 0.72 (0.65–0.84) mm^3^/J for MWA and 0.48 (0.39–0.54) mm^3^/J for LA. HT was significantly correlated with R_AV/E_ of LA (coefficient =  − 0.367, *p* < 0.0001). R_AL/E_ did not differ significantly between MWA and LA (MWA 0.026 mm/J, LA 0.025 mm/J; *p* = 0.957). However, MWA had a greater R_AO/E_ than LA (MWA 0.014 mm/J, LA 0.012 mm/J; *p* < 0.0001). The plateau values of MWA and LA on the ablation orthogonal diameter were 10.7 mm and 8.69 mm, respectively.

**Conclusions:**

MWA showed a higher R_AV/E_ than LA. More intuitively, MWA had a better ablation performance than LA on the orthogonal axis rather than the longitudinal axis. Theoretically, MWA and LA could achieve complete ablation of ≤ 6.70 mm and ≤ 4.69 mm PTMC separately by single-applicator fixed ablation considering a unilateral 2-mm safe margin. HT had a negative effect on LA but not on MWA.

**Clinical relevance statement:**

This study establishes strong connections between ablation energy and ablation range in papillary thyroid microcarcinoma (PTMC) in vivo, possibly contributing to the supplementation of the PTMC Ablation Consensus or Guidelines and providing a scientific basis for choosing clinical ablation parameters in PTMC.

**Key Points:**

• *Both microwave ablation (MWA) and laser ablation (LA) have excellent performance on the ablation longitudinal axis (easily exceeding 10 mm) for papillary thyroid microcarcinoma (PTMC)*.

• *MWA performed much better than LA on the ablation orthogonal axis*.

• *MWA and LA are expected to achieve complete ablation of* ≤ *6.70 mm and* ≤ *4.69 mm PTMC separately by single-applicator fixed ablation considering a unilateral 2-mm safe margin*.

**Supplementary Information:**

The online version contains supplementary material available at 10.1007/s00330-024-10636-4.

## Introduction

Thyroid cancer has been increasing in recent decades and is the ninth most prevalent malignancy worldwide [1, 2]. More than 50% of newly developed thyroid cancers are papillary thyroid microcarcinoma (PTMC) [3]. Although an active surveillance (AS) strategy is recommended for patients with low-risk PTMC by the American Thyroid Association management guidelines [4], many PTMC patients experience great anxiety during AS and ultimately undergo delayed treatment [5]. Surgical resection (SR) is the first-line treatment for PTMC and has definite efficacy with a low recurrence rate [4]. However, SR can seriously affect quality of life (such as scar formation and life-long thyroid hormone replacement) and is also a great psychological burden for patients [6].

Ultrasound-guided thermal ablation, a well-accepted minimally invasive treatment [7, 8], seems to be an intermediate option between AS and SR and even an alternative treatment option versus SR for low-risk PTMC [9, 10]. Mounting research [9, 11, 12], including long‐term (10 years) follow‐up studies, has demonstrated that thermal ablation, including laser ablation (LA), microwave ablation (MWA), and radiofrequency ablation (RFA), is an effective and safe treatment modality for PTMC. Therefore, a growing number of centers are starting thermal ablation treatment strategies for patients with PTMC, and patients are willing to accept this minimally invasive therapy.

However, the implementation of thermal ablation for PTMC is highly experience-dependent and will be a challenge in clinical practice [13]. In particular, the seriously insufficient data on ablation parameters of PTMC further increases the difficulty. Although manufacturers provide algorithms regarding applied energy or ablation time to predict the ablation range and help operators set the ablation parameters, these algorithms are mostly based on ex vivo data of nonperfused and non-thyroid organs. It can be expected that the predicted ablation range would differ substantially from that of the in vivo thyroid [14], which might markedly mislead operators and increase the risk of incomplete ablation or over ablation. Furthermore, whether some PTMC or thyroid characteristics affect thermal ablation remains unclear and should also be considered before implementing ablation [14, 15].

In this study, we aim to (1) explore the relationship between the applied energy of MWA or LA and the resulting ablation range during in vivo PTMC treatment, and (2) compare the ablation characteristics of MWA and LA to provide suggestions for clinical decision-makings.

## Methods

### Patients

**Fig. 1:**
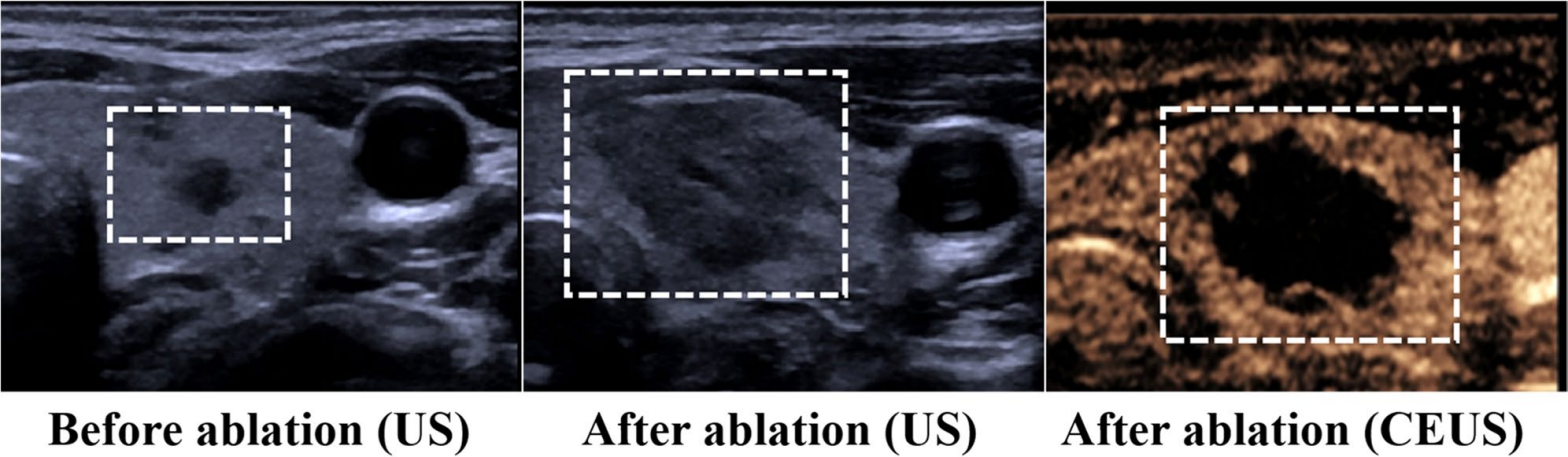
Illustration of single-applicator fixed ablation (MWA, 30 w, 13 s) that achieved full coverage of PTMC (5 × 2.8 × 3 mm) in the ablation area (10.7 × 6.4 × 6.4 mm)

This retrospective study was approved by the Ethics Committee of the Third Xiangya Hospital. Before LA or MWA, patients provided full written informed consent for the treatment. All thermoablation procedures were performed by a radiologist with more than 10 years of experience in US-guided thermal ablation of thyroid nodules. From January 2021 to October 2022, 201 PTMC patients who were treated by MWA or LA were screened for inclusion in this study according to the following criteria. The inclusion criteria were as follows: (1) pathologically confirmed PTMC (diameter ≤ 10 mm) based on fine needle aspiration; (2) no evidence of extrathyroidal extension, capsular infiltration, lymph node metastasis, or distant metastasis on imaging; (3) willingness to receive interventional therapy while refusing surgery; (4) complete ablation achieved by “single-applicator fixed ablation” (Fig. 1); (5) US and CEUS performed before and after ablation. The exclusion criteria were as follows: (1) nodule located in the thyroid isthmus; (2) superimposed ablation in one nodule; (3) two or more ablation zones in the unilateral thyroid lobe; and (4) ablation zones exceeding the margin of the thyroid gland.

### Pre-ablation assessment

Patients received US, CEUS, and thyroid function examinations before MWA or LA. Conventional US was mainly used to record the nodule diameters, position, and ultrasound characteristics, and search for suspicious lymph node metastasis. CEUS was used to evaluate the microvascular perfusion of the nodule. During CEUS, equal/low enhancement was defined when the nodule echogenicity was equal/lower than that of the thyroid parenchyma [16]. The nodule volume (V) was calculated by *V* = π*abc/6, where *a* is the largest diameter of the nodule and *b* and *c* are the two perpendicular diameters.

Hashimoto’s thyroiditis (HT) was diagnosed by combining US imaging (thyroid diffuse hypoechoic parenchyma) and serological examinations (elevated thyroid peroxidase antibody or thyroglobulin antibody).

### Ablation procedure

**Fig. 2:**
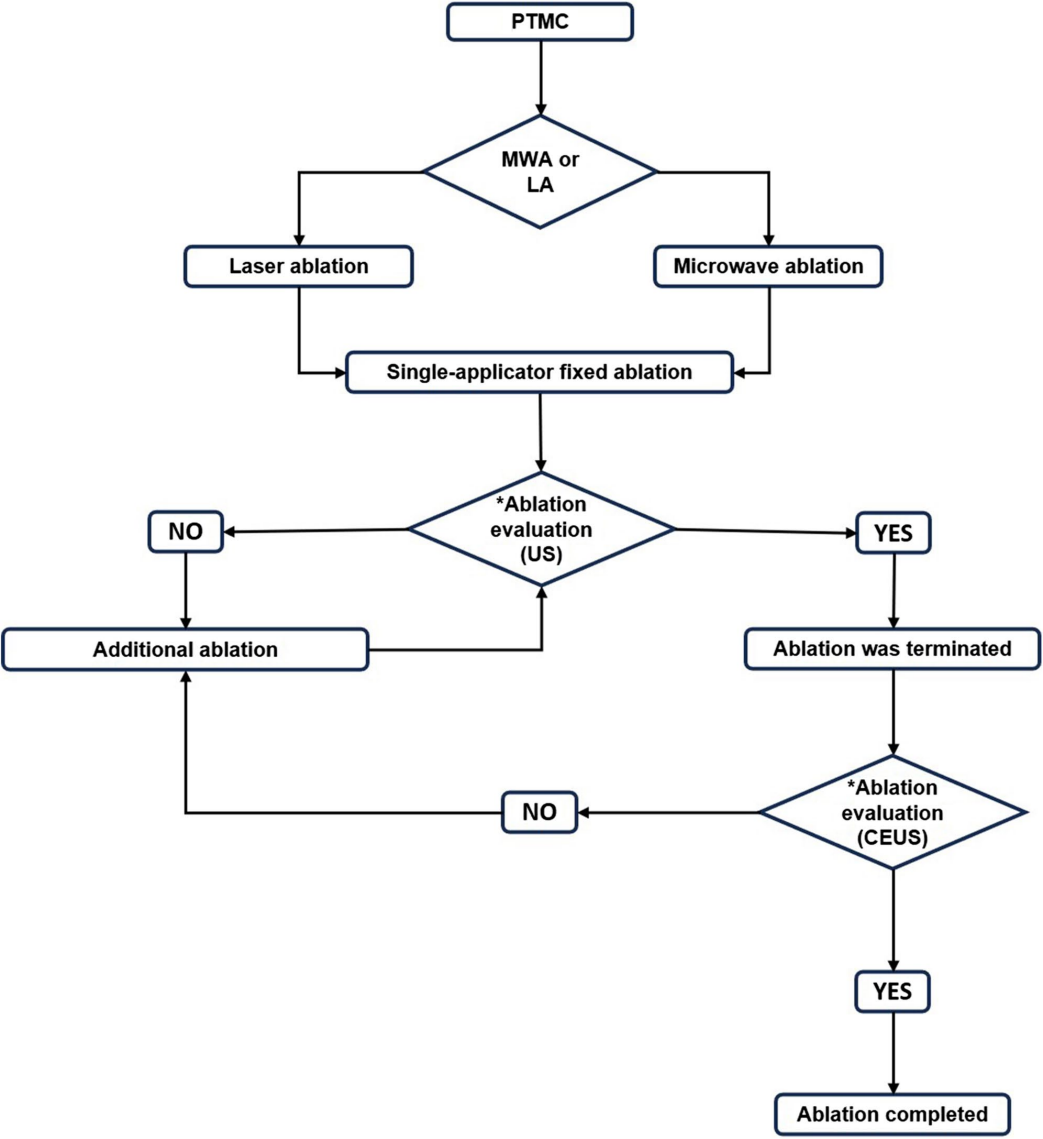
Flowchart on how the clinical decision was made on the ablation technique in the clinic. *Ablation evaluation (US): transient hyperechoic cloud by the ablation covered the whole nodule on US; *ablation evaluation (CEUS): CEUS was performed immediately after ablation to assess whether the nonenhanced zone had covered and exceeded the original nodule on CEUS

The flowchart on how the clinical decision was made on the ablation technique is shown in Fig. 2. The patient lay in the supine position with the neck fully exposed. All operations were performed under aseptic conditions and local anesthesia with 1% lidocaine.

For MWA (KY-2000, Kangyou Medical, 2450 MHz), a 16-G cooled MWA antenna with a 6-mm tip was inserted into the nodule center under US guidance. The power output was maintained at 30 W. MWA was terminated when the transient hyperechoic ablation zone covered the whole nodule. The immediate post-ablation CEUS was performed to confirm complete ablation. The ablation time was recorded, and the applied ablation energy was calculated by ablation power × ablation time.

For LA (EcholaserX4, Elesta, 1064 ± 10 nm laser wavelength), a 21-G trocar was inserted into the center of the nodule under US guidance. After the core needle was removed, a 300-μm optical fiber was inserted into the same position through the sheath of the trocar. The tip of the optical fiber was exposed to contact the nodule directly by retracting the trocar by 5 mm. The power output was maintained at 4 W. The LA was terminated when the transient hyperechoic ablation zone covered the whole nodule. The immediate post-ablation CEUS was performed to confirm complete ablation. The applied ablation energy and ablation time were recorded.

### Ablation range

**Fig. 3:**
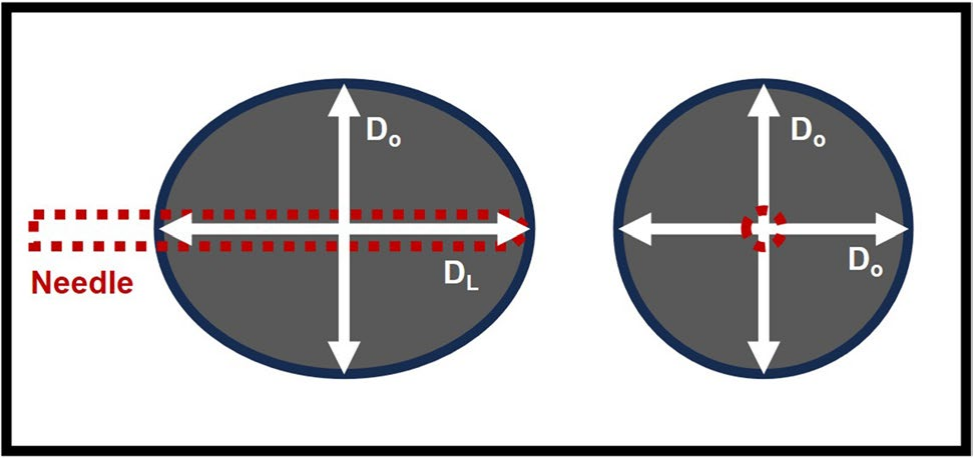
Illustration of the ablation longitudinal diameter and orthogonal diameter by single-applicator fixed ablation. D_L_, longitudinal diameter; D_o_, orthogonal diameter

Contrast-enhanced ultrasound (CEUS) was performed 1 h after the complete ablation to record the ablation range. The ablation zone volume (*V*) was calculated by *V* = π*abc/6, where *a* is the longitudinal diameter of the ablation zone and *b* and *c* are the two orthogonal diameters. The *b* value is approximately equal to the *c* value in single-applicator fixed ablation (Fig. 3). R_AV/E_, R_AL/E_, and R_AO/E_ were calculated by dividing the ablation volume (in cubic millimeters), longitudinal diameter (in millimeters), and orthogonal diameter (in millimeters) by the ablation energy in joules. R_AV/T_ was calculated by dividing the ablation volume in cubic millimeters by the ablation time in seconds.

### Statistical analysis

Data statistical analysis, graph drawing, and curve fitting were performed by SPSS (version 23.0, SPSS) and GraphPad Prism (version 8.0, GraphPad software).

The normality of parameters was tested by the Shapiro-Wilk test. The normally distributed variables are presented as the mean ± standard deviation and were compared through the independent-samples *t* test. The non-normally distributed variables are presented as medians with interquartile ranges (IQRs) and were compared by the Mann-Whitney *U* test. Dichotomous variables were analyzed using Fisher’s exact test. A *p* value < 0.05 was considered indicative of statistical significance.

To compare MWA and LA, propensity score matching was performed to balance the preliminary data and reduce confounding bias. The baseline characteristics were as follows: gender, age, nodule diameter, nodule calcification, CEUS characteristics, HT, and ablation energy. After the 1:1 match, quantitative variables were analyzed by Fisher’s exact test, and quantitative variables were analyzed by *t* test or Mann-Whitney *U* test. A *p* value < 0.05 was considered indicative of statistical significance.

The correlations between R_AV/E_ and PTMC characteristics (position, microcalcification, CEUS) or Hashimoto’s thyroiditis were analyzed by linear regression. A *p* value < 0.05 was considered indicative of statistical significance.

To establish the connection between ablation diameters and ablation energy, ablation orthogonal/longitudinal diameter was defined as *y*, and ablation energy was defined as *x*. The effect of the ablation energy (*x*) on R_AL/E_ or R_AO/E_ (*y*/*x*) was evaluated using Spearman’s rank test. The algorithm “one phase decay” (GraphPad Prism) was used to construct the fitting model between ablation longitudinal/orthogonal diameter (*y*) and ablation energy (*x*).

## Results

### Patient and PTMC characteristics

A total of 201 patients with 201 PTMCs treated by “single-applicator fixed ablation (Fig. 1)” were enrolled in this study. The baseline characteristics are presented in Table 1. Of the 201 patients, 50.7% of patients (102 of 201; mean age 40.9; male/female 26/76) underwent LA and 49.3% (99 of 201; mean age 41.0; male/female 22/76) underwent MWA. The PTMCs in the LA group were smaller than those in the MWA group (nodule diameter, 4.2 ± 1.1 mm vs. 6.2 ± 1.6 mm, *p* < 0.001; nodule volume, 19.0 mm^3^ (11.7–32.5) vs. 72.1 mm^3^ (43.3–118.9), *p* < 0.001). More microcalcification nodules were treated with MWA than LA (*p* = 0.014). Other characteristics showed no difference between the two groups (Table 1).

**Table 1:** Patients and nodule characteristics

	Total	LA	MWA	*p* value
Patients	201	102	99	
Gender (man/female)	48/153	26/76	22/76	
Age	40.9 (10.8)	40.9 (10.2)	41.0 (11.5)	0.970
Number of lesions	201	102	99	
Nodule diameter* (mm)	5.2 (1.7)	4.2 (1.1)	6.2 (1.6)	< 0.001
Nodule volume (mm^3^)	37.4 (17.0–77.3)	19.0 (11.7–32.5)	72.1 (43.3–118.9)	< 0.001
Microcalcification (+ / −)	77/121	31/71	47/52	0.014
Nodule CEUS				
Equal enhancement	76	45	31	0.075
Low enhancement	135	57	68	…
Nodule position				
Left lobe	88	45	43	1.000
Right lobe	113	57	56	…
HT (+ / −)	38/163 (23.3%)	23/79 (29.1%)	15/84 (17.9%)	0.209

*LA* laser ablation, *MWA* microwave ablation, *HT* Hashimoto’s thyroiditis. Nodule diameter*: the largest diameter of the nodule

Normally distributed parameters were presented as mean (SD)

Non-normally distributed parameters were presented as medians (IQR 1–3)

### The relationship between ablation range and ablation energy

Table 2 shows the ablation parameters of MWA and LA. The technical success rates were both 100% and no major complications were found. It is worth noting that the R_N/A_ was remarkably low in both groups (MWA, 15.4%; LA, 9.5%). The applied energy and ablation volume in the MWA group were greater than those in the LA group (MWA 600 J, LA 440 J, *p* < 0.0001; MWA 461.58 mm^3^, LA 196.94 mm^3^, *p* < 0.0001). Nevertheless, the ablation time was significantly shorter for MWA (MWA 20 s, LA 110 s, *p* < 0.0001). Correspondingly, the R_AV/T_ was higher for MWA than for LA (MWA 38.5, LA 3.35, *p* < 0.0001). The ablation efficacy (R_AV/E_, R_AL/E_, R_AO/E_) of MWA and LA is also listed in Table 2.

**Table 2:** The ablation parameters of MWA and LA

	LA	MWA	*p* value
Applied energy (J)	440 (400–500)	600 (510–750)	< 0.0001
Ablation volume (mm^3^)	196.94 (162.27–255.42)	461.58 (359.93–574.72)	< 0.0001
Ablation time (s)	110 (100–125)	20 (17–25)	< 0.0001
R_AV/E_ (mm^3^/J)	0.48 (0.39–0.54)	0.72 (0.65–0.84)	< 0.0001
R_AL/E_ (mm/J)	0.028 (0.025–0.031)	0.023 (0.018–0.027)	< 0.0001
R_AO/E_ (mm/J)	0.012 (0.011–0.014)	0.014 (0.012–0.015)	0.027
R_AV/T_ (mm^3^/s)	1.90 (1.54–2.16)	21.49 (19.38–25.33)	< 0.0001
R_N/A_	9.5% (6.3–19.0%)	15.4% (11.2–24.2%)	< 0.0001
Technical success rate	100%	100%	(-)
Needle diameter	21 G	16 G	(-)

*LA* laser ablation, *MWA* microwave ablation

*R*_*AV/E*_, the ratio of ablation volume to ablation energy

*R*_*AL/E*_, the ratio of ablation longitudinal diameter to ablation energy

*R*_*AO/E*_, the ratio of ablation orthogonal diameter to ablation energy

*R*_*AV/T*_, the ratio of ablation volume to ablation time

*R*_*N/A*_, the ratio of nodule volume to ablation zone volume

Non-normally distributed parameters were presented as medians (IQR 1–3)

#### Comparing ablation efficiency between LA and MWA

**Fig. 4:**
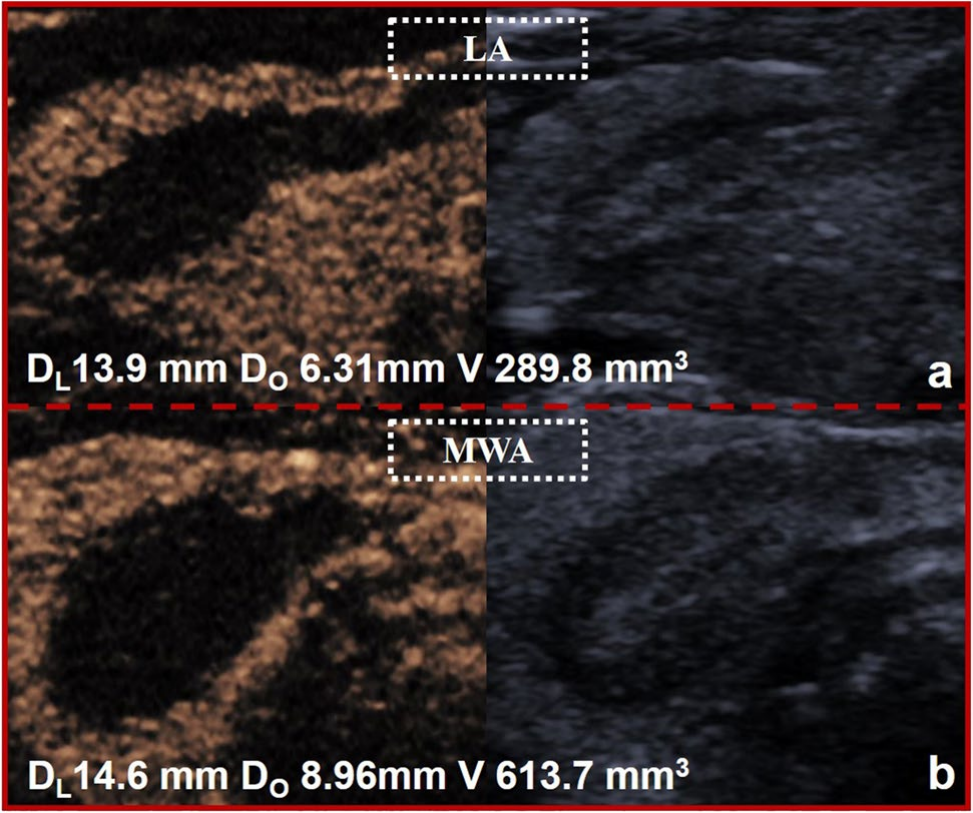
Comparison between LA and MWA using the same energy output. The ablation range was 13.9 × 6.31 × 6.31 mm (volume: 289.8 mm^3^) (**a**) for LA (4 w, 150 s, 600 J) and 14.6 × 8.96 × 8.96 mm (volume: 613.7 mm^3^) (**b**) for MWA (30 w, 20 s, 600 J). D_L_, longitudinal diameter; D_o_, orthogonal diameter; V, ablation volume

To reduce confounding bias, propensity score matching was performed in this study. After 1:1 matching, the baseline characteristics are presented in Supplementary Information Table S1. R_AV/E_, R_AL/E_, and R_AO/E_ were compared between LA and MWA. As shown in Table 3, the R_AV/E_ of MWA was higher than that of LA (*p* < 0.0001). Furthermore, R_AO/E_ was higher for MWA than for LA (*p* < 0.0001), but R_AL/E_ did not differ significantly (*p* = 0.957), indicating that MWA had a better ablation effect than LA on the orthogonal axis but not on the longitudinal axis (Fig. 4).

**Table 3:** The comparison between MWA and LA after propensity score matching

	LA	MWA	*p* value
R_AV/E_ (mm^3^/J)	0.49 (0.38–0.53)	0.69 (0.62–0.78)	< 0.0001
R_AO/E_ (mm/J)	0.012 (0.011–0.014)	0.014 (0.013–0.016)	< 0.0001
R_AL/E_ (mm/J)	0.025 (0.022–0.030)	0.026 (0.023–0.030)	0.957

*LA* laser ablation, *MWA* microwave ablation

*R*_*AV/E*_, the ratio of ablation volume to ablation energy

*R*_*AL/E*_, the ratio of ablation longitudinal diameter to ablation energy

*R*_*AO/E*_, the ratio of ablation orthogonal diameter to ablation energy

Non-normally distributed parameters were presented as medians (IQR 1–3)

#### The potential impact factors on ablation efficiency

As shown in Table 4, there were no significant correlations between R_AV/E_ and PTMC characteristics (such as microcalcification and CEUS blood supply) in either group. However, HT had a negative effect on R_AV/E_ for LA (− 0.367, *p* < 0.001) but not for MWA (− 0.045, *p* = 0.669). Furthermore, the effect of HT on R_AL/E_ or R_AO/E_ of LA was also recorded (Supplementary Information Table S2).

**Table 4:** The effect of patients’ and nodules’ characteristics on R_AV/E_

	LA		MWA	
	*S*-coefficient	*p* value	*S*-coefficient	*p* value
Gender (man/female)	− 0.114	0.262	− 0.039	0.704
Age	− 0.046	0.654	− 0.087	0.417
HT (+ / −)	− 0.367	** < 0.0001**	− 0.045	0.669
Nodule position	− 0.136	0.161	0.175	0.097
Nodule volume	− 0.150	0.168	− 0.126	0.243
Nodule calcification (+ / −)	0.038	0.716	− 0.141	0.178
Nodule CEUS*	0.064	0.648	− 0.026	0.801

*LA* laser ablation, *MWA* microwave ablation, *HT* Hashimoto’s thyroiditis, *S-coefficient* standardized coefficient

*R*_*AV/E*_, the ratio of ablation volume to ablation energy

Nodule CEUS*, equal enhancement (+) and low enhancement (−)

### Establish fitting models between ablation energy and ablation diameters

As mentioned above, HT had a negative effect on LA. Considering that it is difficult to quantify the degree of inflammation, the patients with HT in the LA group were excluded from subsequent research.

**Fig. 5:**
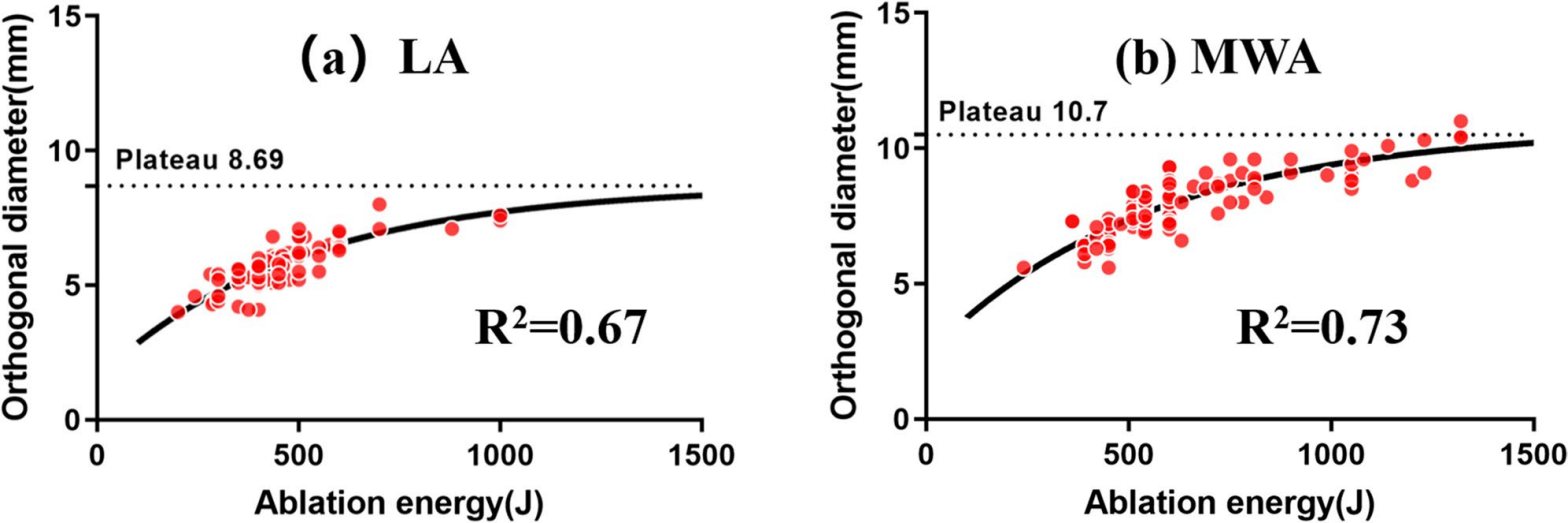
The fitting curve based on the ablation orthogonal diameter and ablation energy of LA (**a**) and MWA (**b**) using the “one phase decay” algorithm

The R_AO/E_ was correlated with ablation energy with a Spearman’s correlation coefficient of − 0.892 for MWA (*p* < 0.0001) and − 0.742 for LA (*p* < 0.0001), indicating that larger ablation energy resulted in a lower R_AO/E_. The fitting models between the ablation orthogonal diameter (*y*) and ablation energy (*x*) are shown in Fig. 5, and the plateau values of MWA and LA were 10.7 mm (*R* square = 0.73) and 8.69 mm (*R* square = 0.67), respectively, which represented the theoretical maximum ablation diameter on the orthogonal axis by single-applicator fixed ablation.

**Fig. 6:**
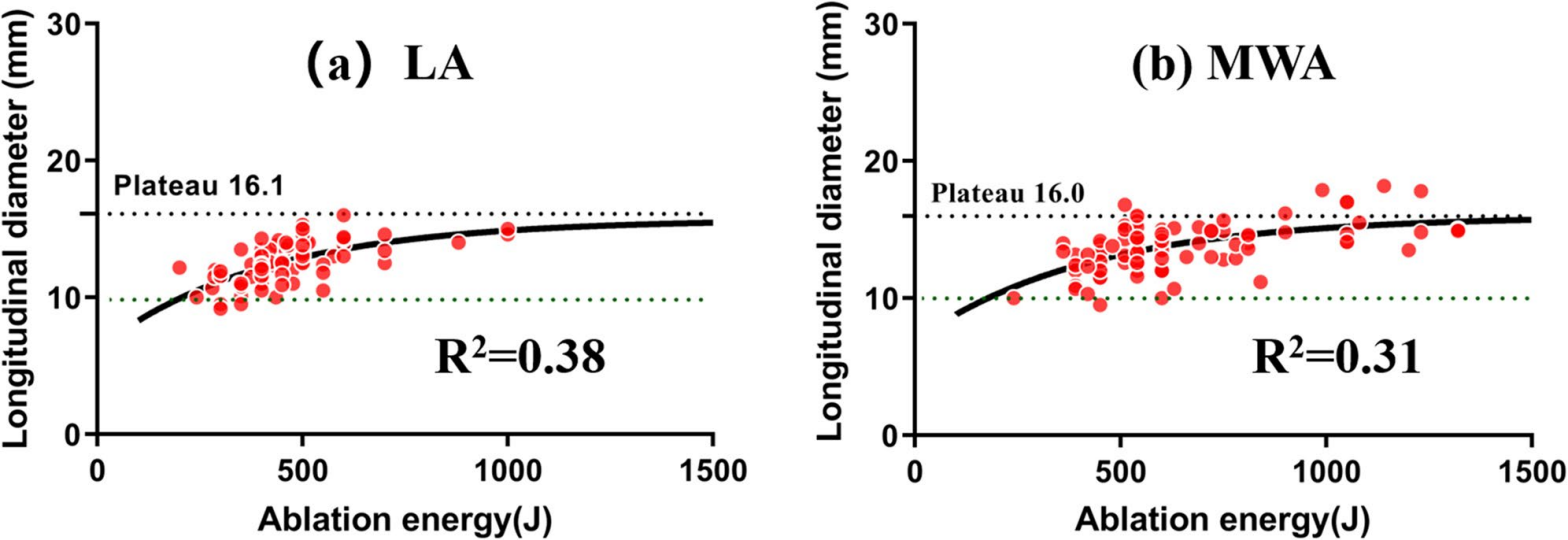
The fitting curve based on the ablation longitudinal diameter and ablation energy of LA (**a**) and MWA (**b**) and using the “one phase decay” algorithm

The R_AL/E_ also had a negative correlation with ablation energy: a Spearman’s correlation coefficient of − 0.867 for MWA (*p* < 0.0001) and − 0.791 for LA (*p* < 0.0001), indicating that larger ablation energy resulted in a lower R_AL/E_. The fitting models between the ablation longitudinal diameter (*y*) and ablation energy (*x*) are shown in Fig. 6. Clearly, both MWA and LA had excellent performance on the ablation longitudinal axis because the ablation longitudinal diameter could easily exceed 10 mm (Fig. 6), while PTMC is defined as small papillary thyroid carcinoma with greatest dimension ≤ 10 mm.

## Discussion

Thermal ablation has been regarded as an effective minimally invasive treatment for PTMC and has gained growing popularity worldwide. However, implementing thermal ablation for PTMC is challenging, especially because the thyroid is small and closely adjacent to many essential structures. Therefore, it is extremely important to know the relationship between the ablation range and ablation energy when choosing an appropriate ablation method, ablation technique, and ablation energy in PTMC.

To explore the relationship between ablation range and ablation energy as precisely as possible, only PTMCs that were inactivated by “single-applicator fixed ablation” were included in this study. The results showed that the R_AV/E_ was 0.72 (0.65–0.84) mm^3^/J for MWA and 0.48 (0.39–0.54) mm^3^/J for LA. After propensity score matching, the R_AV/E_ of MWA was significantly greater than that of LA (*p* < 0.0001). Upon further analysis, we found that MWA had a better ablation performance on the ablation orthogonal axis (*p* < 0.0001), which could be explained by their different working principles. It has been demonstrated that the longer the wavelength of electromagnetic radiation, the deeper the penetration depth [17]. Therefore, compared to LA with a short wavelength of 1064 nm, a longer microwave wavelength (up to 122 mm) endows MWA with a stronger penetrating ability, which could heat the target tissue quickly and evenly and readily penetrate through the charred tissues around the ablation applicators. Regarding the longitudinal axis, both MWA and LA had excellent performance (easily exceeding 10 mm), and R_AL/E_ did not differ significantly between MWA and LA (*p* = 0.957). Active ablation tips with similar lengths (MWA, 6 mm; LA, 5 mm) might explain this.

Ablation longitudinal and orthogonal diameters are important intuitive parameters for operators during the ablation procedure. In fact, the evaluation and prediction of ablation ability on the orthogonal axis are much more important than those on the ablation longitudinal axis. The ablation longitudinal diameter can readily be over 10 mm and be further extended through a “pull back way [18]”. In addition, the ablation range is often clearly visualized by hyperechogenicity clouds during thermal ablation on the longitudinal axis but not on the ablation orthogonal axis due to acoustic shadow interference. Therefore, we established the fitting model based on ablation orthogonal diameter (*y*) and ablation energy (*x*) for MWA [*y* =  − 8.394*exp(− 0.001826*x*) + 10.74, *R*^2^ = 0.73] and for LA [*y* =  − 7.125*exp(− 0.001992*x*) + 8.693, *R*^2^ = 0.67]. It is worth noting that the “plateau value” that represented the theoretical maximum ablation orthogonal diameter was 10.7 mm for MWA and 8.69 mm for LA. In other words, MWA and LA could achieve complete ablation of ≤ 6.7 mm and ≤ 4.69 mm PTMC by single-applicator fixed ablation because the ablation range along the orthogonal axis (the shorter one of ablation diameters) could encompass the maximum diameter of the PTMC and exceed a unilateral 2-mm safe margin.

The potential impact factors on the ablation range were also investigated in this study. Our results showed that the R_AV/E_ was not affected by PTMC characteristics, such as microcalcification and blood supply (CEUS). This could be attributed to the fact that the thyroid parenchyma, but not the PTMC, takes up the major component of ablated tissue because the ablation zone must cover and exceed the PTMC with a safe margin [18–20]. Therefore, the low R_N/A_ could explain the finding that R_AV/E_ was not affected by PTMC characteristics, which is consistent with the studies by Heerink et al [21] for liver tumors and Deshazer et al [22] for phantom models. Instead, the property of peritumor tissue parenchyma was found to be the predominant impact factor on the ablation range [22]. Indeed, we found that Hashimoto’s thyroiditis was a negative impact factor for LA. Compared to a normal thyroid, HT is often accompanied by fibrosis and increased blood supply of the thyroid parenchyma [23]. Thyroid fibrous tissue can exert an “oven effect” and cause low thermal conductivity [24]. In addition, the rich thyroid blood supply could accelerate heat loss [25]. However, MWA not only relies less on thermal conductivity than LA, but is also insusceptible to the “heat sink” effect due to its shorter ablation time and faster heating rate [26]. This explains our findings that Hashimoto’s thyroiditis had no significant effect on MWA. Thus, we advise MWA for PTMC in thyroid with HT rather than LA.

Our findings fill the gap between MWA and LA ablation parameters in PTMC in vivo and may provide critical information for clinical decision-making. In summary, both MWA and LA have excellent performance on the ablation longitudinal axis (easily exceeding 10 mm). However, MWA has better ablation performance on the orthogonal axis than LA. Theoretically, MWA and LA could achieve complete ablation of ≤ 6.7 mm and ≤ 4.69 mm PTMC separately by single-applicator fixed ablation considering a unilateral 2-mm safe margin. Therefore, we advise MWA rather than LA for > 4.69 mm and ≤ 6.70 mm PTMC. However, considering the unique advantages of LA on safe and precise energy output and lower complication rate [27], we think LA could be a better choice for ≤ 4.69 mm PTMC. For larger PTMC (> 6.7 mm), superimposed ablation is advised. In addition, MWA but not LA is advised for PTMC in thyroid with HT background.

### Supplementary Information

Below is the link to the electronic supplementary material.

Supplementary file1 (PDF 120 KB)

## Abbreviations

AS: Active surveillance
CEUS: Contrast-enhanced ultrasound
HT: Hashimoto’s thyroiditis
IQRs: Interquartile ranges
LA: Laser ablation
MWA: Microwave ablation
PTMC: Papillary thyroid microcarcinoma
R_AV/E_: The ratio of ablation zone volume to ablation energy
R_AL/E_: The ratio of ablation longitudinal diameter to ablation energy
R_AO/E_: The ratio of ablation orthogonal diameter to ablation energy
R_AV/T_: The ratio of ablation zone volume to ablation time
R_N/A_: The ratio of nodular volume to ablation zone volume
SR: Surgical resection
US: Ultrasound

## Declarations

### Guarantor

The scientific guarantor of this publication is Ping Zhou.

### Statistics and biometry

One of the authors has significant statistical expertise.

### Study subjects or cohorts overlap

No overlap.

### Methodology

• retrospective

• observational

• performed at one institution
