# Supplementary material for: The relationship between ablation range and ablation energy in papillary thyroid microcarcinoma: a comparison between microwave ablation and laser ablation
Source: Eur Radiol. 2024 Feb 10;34(9):6072–81. doi: 10.1007/s00330-024-10636-4 (PMC11364595; doi:10.1007/s00330-024-10636-4)
Supplement: Supplementary file 1 — Supplementary file1 (PDF 120 KB) [file 330_2024_10636_MOESM1_ESM.pdf]

The relationship between ablation range and ablation energy in papillary thyroid microcarcinoma: A comparison between microwave ablation and laser ablation

ELECTRONIC SUPPLEMENTARY MATERIAL

Table S1 Patients and nodule characteristics after propensity-Score Matching

	Total	LA	MWA	P value
Patients	64	32	32	
Gender (man/female)	19/45	11/21	8/24	0.585
Age	42.0 (11.4)	40.7 (10.8)	43.2 (12.0)	0.386
Number of lesions	64	32	32	
Nodule diameter (mm)	5.0 (1.19)	5.0 (1.21)	5.0 (1.19)	0.975
Microcalcification (+/-)	26/38	12/20	14/18	0.799
**Nodule CEUS**				
Equal enhancement	23	12	11	1.000
Low enhancement	41	20	21	
**Nodule position**				
Left lobe	29	16	13	0.616
Right lobe	35	16	19	
**HT (+/-)**	12/52	7/25	5/27	0.750
**Ablation energy**	510 (405-600)	455 (400-600)	525 (428-600)	0.539

LA laser ablation, MWA microwave ablation, HT Hashimoto's thyroiditis.

Normally distributed parameters were presented as mean (SD).

Non-normally distributed parameters were presented as medians (IQR 1-3).

Table S2: The correlations between Hashimoto's thyroiditis (HT) and R values

The effect of HT to $R_{AO/E}$

	LA ($R_{AO/E}$)		MWA ($R_{AO/E}$)	
	S-Coefficient	P value	S-Coefficient	P value
HT (+/-)	-0.315	0.001	-0.065	0.521

The effect of HT to $R_{AL/E}$

	LA ($R_{AL/E}$)		MWA ($R_{AL/E}$)	
	S-Coefficient	P value	S-Coefficient	P value
HT (+/-)	-0.136	0.172	-0.078	0.445

LA laser ablation, MWA microwave ablation, HT Hashimoto's thyroiditis, S-Coefficient: Standardized Coefficient.

$R_{AO/E}$: the ratio of ablation orthogonal diameter to ablation energy.

$R_{AL/E}$: the ratio of ablation longitudinal diameter to ablation energy.
